# Supplementary material for: Association of premature menopause with incident pulmonary hypertension: A cohort study
Source: PLoS One. 2021 Mar 10;16(3):e0247398. doi: 10.1371/journal.pone.0247398 (PMC7946190; doi:10.1371/journal.pone.0247398)
Supplement: S1 Checklist — (DOCX) [file pone.0247398.s001.docx]

**S1 Checklist.**

|  | Item No | Recommendation | Page  No |
| --- | --- | --- | --- |
| **Title and abstract** | 1 | (*a*) Indicate the study’s design with a commonly used term in the title or the abstract ***Association of premature menopause with incident pulmonary hypertension: A cohort study*** | 1 |
|  |  | (*b*) Provide in the abstract an informative and balanced summary of what was done and what was found | 3-4 |
| Introduction | | | |
| Background/rationale | 2 | Explain the scientific background and rationale for the investigation being reported ***Animal and human studies suggest that sex hormones influence PH development, although observed effects of estrogen and its metabolites have been conflicting across and within animal models… [D]ata on the relationship of menopause to PH are limited (8), and whether premature menopause represents a risk factor for PH is unknown.*** | 5 |
| Objectives | 3 | State specific objectives, including any prespecified hypotheses ***Here, we tested the association of premature menopause with incident PH among postmenopausal women in the UK Biobank. We hypothesized that premature menopause would be independently associated with increased risk of PH.*** | 5 |
| Methods | | | |
| Study design | 4 | Present key elements of study design early in the paper | 5-6 |
| Setting | 5 | Describe the setting, locations, and relevant dates, including periods of recruitment, exposure, follow-up, and data collection ***The UK Biobank is a population-based cohort that includes >500,000 adult residents of the United Kingdom who were recruited between 2006-2010. … Follow-up in the UK Biobank is performed on an ongoing basis through study visits and linkage to national health records and the death register, as described previously (7).*** ***In the present analysis, follow-up in the UK Biobank occurred through March 2020 for inpatient diagnosis codes and May 2020 for the death register.*** | 5-6 |
| Participants | 6 | (*a*) *Cohort study*—Give the eligibility criteria, and the sources and methods of selection of participants. Describe methods of follow-up  *Case-control study*—Give the eligibility criteria, and the sources and methods of case ascertainment and control selection. Give the rationale for the choice of cases and controls  *Cross-sectional study*—Give the eligibility criteria, and the sources and methods of selection of participants ***Follow-up is performed on an ongoing basis through study visits and linkage to national health records and the death register, as described previously (7).*** … ***In this cohort study, all postmenopausal women aged 40-69 years old with complete reproductive data were considered for inclusion. Women with baseline PH, congenital heart disease, or extreme pulmonary function indices (Z>5 or Z<-5) were excluded.*** | 6 |
|  |  | (*b*) *Cohort study*—For matched studies, give matching criteria and number of exposed and unexposed  *Case-control study*—For matched studies, give matching criteria and the number of controls per case | NA |
| Variables | 7 | Clearly define all outcomes, exposures, predictors, potential confounders, and effect modifiers. Give diagnostic criteria, if applicable ***The primary exposure was premature menopause (before age 40 years), ascertained by participant self-report at study enrollment. The study outcome was incident PH, ascertained by the appearance of a qualifying ICD code (ICD-9 4160; ICD-10 I27.0, I27.2) in the study record….*** ***Cox proportional hazard models tested the association of premature menopause with incident PH, with adjustment for age, race/ethnicity, ever-smoking, body-mass index, systolic blood pressure, non-high-density lipoprotein cholesterol, antihypertensive and cholesterol-lowering medication, log-transformed C-reactive protein, type 2 diabetes mellitus, obstructive sleep apnea (OSA), heart failure, aortic stenosis, mitral regurgitation, venous thromboembolism, FVC, FEV_1_-to-FVC ratio, ever-use of menopausal hormone therapy (MHT), and hysterectomy status.*** | 6 |
| Data sources/ measurement | 8* | For each variable of interest, give sources of data and details of methods of assessment (measurement). Describe comparability of assessment methods if there is more than one group ***Follow-up in the UK Biobank is performed on an ongoing basis through study visits and linkage to national health records and the death register, as described previously (7). In the present analysis, follow-up occurred through March 2020 for inpatient diagnosis codes and May 2020 for the death register.*** | 6 |
| Bias | 9 | Describe any efforts to address potential sources of bias **The UK Biobank is a population-based cohort study with previously described healthy participant bias. We acknowledge this in the discussion of limitations: *“Healthy participant bias” in the UK Biobank (12) may bias the magnitude of estimated associations toward the null.*** | 9 |
| Study size | 10 | Explain how the study size was arrived at **All eligible subjects were included, as noted: *In this cohort study, all postmenopausal women aged 40-69 years old with complete reproductive data were considered for inclusion. Women with baseline PH, congenital heart disease, or extreme pulmonary function indices (Z>5 or Z<-5) were excluded.*** | 6 |
| Quantitative variables | 11 | Explain how quantitative variables were handled in the analyses. If applicable, describe which groupings were chosen and why ***Cox proportional hazard models tested the association of premature menopause with incident PH, with adjustment for age, race/ethnicity, ever-smoking, body-mass index, systolic blood pressure, non-high-density lipoprotein cholesterol, antihypertensive and cholesterol-lowering medication, log-transformed C-reactive protein, type 2 diabetes mellitus, obstructive sleep apnea (OSA), heart failure, aortic stenosis, mitral regurgitation, venous thromboembolism, FVC, FEV_1_-to-FVC ratio, ever-use of menopausal hormone therapy (MHT), and hysterectomy status.*** | 6 |
| Statistical methods | 12 | (*a*) Describe all statistical methods, including those used to control for confounding ***Cox proportional hazard models tested the association of premature menopause with incident PH, with adjustment for age, race/ethnicity, ever-smoking, body-mass index, systolic blood pressure, non-high-density lipoprotein cholesterol, antihypertensive and cholesterol-lowering medication, log-transformed C-reactive protein, type 2 diabetes mellitus, obstructive sleep apnea (OSA), heart failure, aortic stenosis, mitral regurgitation, venous thromboembolism, FVC, FEV_1_-to-FVC ratio, ever-use of menopausal hormone therapy (MHT), and hysterectomy status.*** | 6 |
|  |  | (*b*) Describe any methods used to examine subgroups and interactions | NA |
|  |  | (*c*) Explain how missing data were addressed ***In primary models, subjects with missing data were excluded. Multiple sensitivity analyses were performed, including analyses with imputed missing covariates using the predict() function in R based on age, race/ethnicity, and premature menopause status.*** | 6 |
|  |  | (*d*) *Cohort study*—If applicable, explain how loss to follow-up was addressed  *Case-control study*—If applicable, explain how matching of cases and controls was addressed  *Cross-sectional study*—If applicable, describe analytical methods taking account of sampling strategy ***Subjects were followed until a diagnosis of PH or until their last clinical encounter or study visit.*** | 6 |
|  |  | (*e*) Describe any sensitivity analyses ***Multiple sensitivity analyses were performed, including analyses with imputed missing covariates using the predict() function in R based on age, race/ethnicity, and premature menopause status; analyses excluding women with prevalent cancer and history of hysterectomy; analyses excluding women with prevalent heart failure, aortic stenosis, mitral regurgitation, venous thromboembolism, OSA, and/or COPD; and analyses requiring ≥2 instances of ICD coding to classify incident PH.*** | 6 |

Continued on next page

| Results | | | |
| --- | --- | --- | --- |
| Participants | 13* | (a) Report numbers of individuals at each stage of study—eg numbers potentially eligible, examined for eligibility, confirmed eligible, included in the study, completing follow-up, and analysed ***See Figure 1*** | ***Figure 1*** |
|  |  | (b) Give reasons for non-participation at each stage | NA |
|  |  | (c) Consider use of a flow diagram ***See Figure 1*** | ***Figure 1*** |
| Descriptive data | 14* | (a) Give characteristics of study participants (eg demographic, clinical, social) and information on exposures and potential confounders  ***See Table 1*** | ***Table 1*** |
|  |  | (b) Indicate number of participants with missing data for each variable of interest ***Missing data are summarized in Supplemental Table 1.*** | ***Supplemental Table 1*** |
|  |  | (c) *Cohort study*—Summarise follow-up time (eg, average and total amount) ***Over a median 11.1 (interquartile range 10.5-11.8) years of follow-up…*** | 7 |
| Outcome data | 15* | *Cohort study*—Report numbers of outcome events or summary measures over time ***…incident PH was diagnosed in 447 women (0.33%), including 38 (0.73%) with premature menopause and 409 (0.31%) without. Incidence rates were 6.6/10,000 (4.5-8.6/10,000) person-years among women with premature menopause and 2.8/10,000 (95% CI 2.5-3.1/10,000) person-years among women without (difference +3.8/10,000 [95% CI 1.7-5.9/10,000] person-years, P<0.001).*** | 7 |
|  |  | *Case-control study—*Report numbers in each exposure category, or summary measures of exposure | NA |
|  |  | *Cross-sectional study—*Report numbers of outcome events or summary measures | NA |
| Main results | 16 | (*a*) Give unadjusted estimates and, if applicable, confounder-adjusted estimates and their precision (eg, 95% confidence interval). Make clear which confounders were adjusted for and why they were included ***…incident PH was diagnosed in 447 women (0.33%), including 38 (0.73%) with premature menopause and 409 (0.31%) without. Incidence rates were 6.6/10,000 (4.5-8.6/10,000) person-years among women with premature menopause and 2.8/10,000 (95% CI 2.5-3.1/10,000) person-years among women without (difference +3.8/10,000 [95% CI 1.7-5.9/10,000] person-years, P<0.001). After adjustment only for age, premature menopause was associated with a hazard ratio (HR) of 2.51 for incident PH (95% CI 1.80-3.50, P<0.001). After multivariable adjustment, premature menopause was independently associated with PH (HR 2.13, 95% CI 1.31-3.23, P<0.001).*** | 7-8 |
|  |  | (*b*) Report category boundaries when continuous variables were categorized | NA |
|  |  | (*c*) If relevant, consider translating estimates of relative risk into absolute risk for a meaningful time period ***See (a) above*** | 7 |
| Other analyses | 17 | Report other analyses done—eg analyses of subgroups and interactions, and sensitivity analyses ***Associations with PH were similar in sensitivity analyses (1) using imputed data to replace missing covariates (HR 2.15, 95% CI 1.50-3.10, P<0.001); (2) excluding 14,441 women with history of cancer or unknown cancer history (HR 1.84, 95% CI 1.13-2.79, P=0.01); (3) excluding 13,699 women with history of hysterectomy (HR 1.81, 95% CI 0.99-3.32, P=0.05); (4) excluding 6,769 women with prevalent heart failure, aortic stenosis, mitral regurgitation, venous thromboembolism, OSA, and/or COPD (HR 2.52, 95% CI 1.59-4.00, P<0.001); and (5) requiring ≥2 instances of ICD coding to classify incident PH (HR 2.65, 95% CI 1.38-5.07, P=0.003). In analyses of alternate menopausal age thresholds, risk of PH appeared to increase progressively with younger age at menopause (P_trend_ <0.001) (Figure 3). Compared with women who experienced menopause after age 50 years, the HR associated with menopause before age 30 years was 4.82 (95% CI 1.82-12.74, P=0.002).*** | 8 |
| Discussion | | | |
| Key results | 18 | Summarise key results with reference to study objectives ***In a large cohort of postmenopausal women, menopause before age 40 years was independently associated with 2-fold risk of PH. Further increase in PH risk was observed with progressively earlier age at menopause. Higher prevalence of heart failure, valvular heart disease, OSA, COPD, and venous thromboembolism among women with premature menopause did not explain the observed increased risk for PH.*** | 9 |
| Limitations | 19 | Discuss limitations of the study, taking into account sources of potential bias or imprecision. Discuss both direction and magnitude of any potential bias ***This study has limitations. Incident PH diagnoses were ascertained from ICD codes rather than invasive hemodynamics; primary analyses therefore tested associations with overall PH rather than PH subtypes (e.g., PAH vs. secondary PH). Data on MHT doses and preparations were unavailable. “Healthy participant bias” in the UK Biobank (12) may bias the magnitude of estimated associations toward the null. More than 95% of our sample was White, and whether results generalize to diverse populations requires further study. Finally, we were unable to assess the role of estradiol as levels were unavailable in 95% of the cohort (mainly due to levels falling below the assay’s reportable limit in the UK Biobank).*** | 9-10 |
| Interpretation | 20 | Give a cautious overall interpretation of results considering objectives, limitations, multiplicity of analyses, results from similar studies, and other relevant evidence ***Premature menopause may represent an independent risk factor for PH. Further investigation of the role of sex hormones in PH is needed to elucidate pathobiology and identify novel therapeutic targets.*** | 10 |
| Generalisability | 21 | Discuss the generalisability (external validity) of the study results ***More than 95% of the study sample was White, and whether results generalize to diverse populations requires further study.*** | 10 |
| Other information | | | |
| Funding | 22 | Give the source of funding and the role of the funders for the present study and, if applicable, for the original study on which the present article is based ***Funding: Dr. Honigberg is supported by the U.S. National Heart, Lung, and Blood Institute [T32HL094301-07]. Dr. Lahm is supported by grants from the U.S. National Heart, Lung, and Blood Institute [R01HL144727-01A1] and the U.S. Department of Veterans Affairs [VA Merit Award 1I01BX002042-07]. Dr. Ho is supported by the U.S. National Heart, Lung, and Blood Institute [R01HL134893, R01HL140224]. Dr. Natarajan is supported by grants from the U.S. National Heart, Lung, and Blood Institute [R01HL1427, R01HL148565, and R01HL148050], Fondation Leducq [TNE-18CVD04], and a Hassenfeld award from the Massachusetts General Hospital.*** | 11 |

*Give information separately for cases and controls in case-control studies and, if applicable, for exposed and unexposed groups in cohort and cross-sectional studies.

**Note:** An Explanation and Elaboration article discusses each checklist item and gives methodological background and published examples of transparent reporting. The STROBE checklist is best used in conjunction with this article (freely available on the Web sites of PLoS Medicine at http://www.plosmedicine.org/, Annals of Internal Medicine at http://www.annals.org/, and Epidemiology at http://www.epidem.com/). Information on the STROBE Initiative is available at www.strobe-statement.org.
