# Supplementary material for: Association of premature menopause with incident pulmonary hypertension: A cohort study
Source: PLoS One. 2021 Mar 10;16(3):e0247398. doi: 10.1371/journal.pone.0247398 (PMC7946190; doi:10.1371/journal.pone.0247398)
Supplement: S1 Table — (DOCX) [file pone.0247398.s002.docx]

**S1 Table. Summary of data missingness in the study cohort.**

| **Variable** | **Missingness in the dataset** |
| --- | --- |
| Systolic blood pressure | 7,597 (5.6%) |
| Body-mass index | 1,799 (1.3%) |
| Non-high-density lipoprotein cholesterol | 19,648 (14.4%) |
| C-reactive protein | 8,351 (6.1%) |
| Current or former use of hormone therapy | 271 (0.2%) |
